# Targeting stanniocalcin‐1‐expressing tumor cells elicits efficient antitumor effects in a mouse model of human lung cancer

**DOI:** 10.1002/cam4.3852

**Published:** 2021-04-07

**Authors:** Kotaro Abe, Masahiko Kanehira, Shinya Ohkouchi, Sakiko Kumata, Yamato Suzuki, Hisashi Oishi, Masafumi Noda, Akira Sakurada, Eisaku Miyauchi, Tohru Fujiwara, Hideo Harigae, Yoshinori Okada

**Affiliations:** ^1^ Department of Thoracic Surgery Institute of Development, Aging and Cancer Tohoku University Sendai Japan; ^2^ Center for Life Science Research University of Yamanashi Chuo Japan; ^3^ Department of Occupational Health Tohoku University Graduate School of Medicine Sendai Japan; ^4^ Department of Respiratory Medicine Tohoku University Graduate School of Medicine Sendai Japan; ^5^ Department of Hematology and Rheumatology Tohoku University Hospital Sendai Japan

**Keywords:** bystander effect, lung cancer, stanniocalsin‐1 (STC‐1), suicide gene

## Abstract

Lung cancer is the most common cause of cancer‐related death in developed countries; therefore, the generation of effective targeted therapeutic regimens is essential. Recently, gene therapy approaches toward malignant cells have emerged as attractive molecular therapeutics. Previous studies have indicated that stanniocalcin‐1 (STC‐1), a hormone involved in calcium and phosphate homeostasis, positively regulates proliferation, apoptosis resistance, and glucose metabolism in lung cancer cell lines. In this study, we investigated if targeting STC‐1 in tumor cells could be a promising strategy for lung cancer gene therapy. We confirmed that STC‐1 levels in peripheral blood were higher in lung cancer patients than in healthy donors and that STC‐1 expression was observed in five out of eight lung cancer cell lines. A vector expressing a suicide gene, uracil phosphoribosyltransferase (UPRT), under the control of the STC‐1 promoter, was constructed (p*P*_STC‐1_‐UPRT) and transfected into three STC‐1‐positive cell lines, PC‐9, A549, and H1299. When stably transfected, we observed significant cell growth inhibition using 5‐fluorouracil (5‐FU) treatment. Furthermore, growth of the STC‐1‐negative lung cancer cell line, LK‐2 was significantly arrested when combined with STC‐1‐positive cells transfected with p*P*_STC‐1_‐UPRT.

We believe that conferring cytotoxicity in STC‐1‐positive lung cancer cells using a suicide gene may be a useful therapeutic strategy for lung cancer.

## Graphical abstract

In this study, we observed that sera collected from lung cancer patients contained higher level of stanniocalcin‐1 (STC‐1), a hormone maintaining mineral homeostasis, than those from healthy donors. In addition, we showed that effective anti‐tumor effect was obtained by targeting lung cancer cells which express STC‐1. This study elucidated that STC‐1 could be useful as both a molecular target and as a marker for lung cancer.

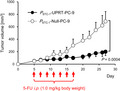

## 1 INTRODUCTION

Globally, lung cancer is a leading cause of death; the disease is widely acknowledged as having extremely poor prognoses thanks to difficulties in achieving complete remission. While surgical resection is the most effective treatment for lung cancer patients at early stages (mainly I and II), systemic treatments such as chemotherapy, radiotherapy, or combinations are the main therapeutic options for lung cancer patients at advanced stages (mainly III and IV). Recently, molecular therapeutics targeting receptor tyrosine kinase (RTK) aberrations in lung cancer, including epidermal growth factor receptor (EGFR) and anaplastic lymphoma kinase (ALK), and immunotherapies have been developed.^1^ These advancements have drastically improved the therapeutic outcomes of some lung cancer types. Nevertheless, drug resistance and disease recurrence are frequently observed, and consequently, the 5‐year survival rate remains poor (<30%). Therefore, novel therapeutic strategies are required to complement or replace existing modalities.

Gene therapy is a promising complementary, alternative treatment strategy for many cancers, with several approaches developed in this area. For example, normal gene insertion into tumor cells to replace a mutant gene (e.g., p53), infection with oncolytic viruses to selectively replicate and lyse tumor cells (e.g., replication‐deficient adenovirus), and the tumor‐specific expression of suicide genes which convert inactive pro‐drugs into active drugs (e.g., herpes virus thymidine kinase [HSV‐tk] combined with ganciclovir [GCV]).^2^ Of these approaches, suicide gene therapy has attracted considerable attention in terms of its safety toward normal cells and targeted efficacy toward tumor cells, thanks to the precise selection of cancer‐specific promoters for suicide gene transcription.

Several promoters have been assessed in a number of suicide gene therapies for cancer. One such promoter originates from the human telomerase reverse transcriptase (hTERT) gene, which is normally expressed in fetal and tumor cells only. It was previously reported that HSV‐tk expression under the control of the hTERT promoter, conferred GCV sensitivity to lung cancer cell lines but not normal cells, and significantly induced apoptosis in lung cancer cells upon GCV treatment in vitro.^3^ In previous studies, the prognostic lung cancer biomarkers, carcinoembryonic antigen (CEA) and squamous cell carcinoma antigen (SCC) were used in suicide gene therapy strategies for lung cancer.^4^, ^5^ The promoter regions of both genes were determined, and their therapeutic potential as suicide gene therapies for lung cancer were examined. However, more research is required in terms of tighter suicide gene regulation/expression, and efficacy for clinical use.

Stanniocalcin‐1 (STC‐1) is a hormone derived from the Stannius bodies of bony fish kidneys, but it also exists in several mammalian species. Secreted as a glycoprotein, the hormone maintains calcium and phosphate homeostasis by controlling phosphate transport across epithelia in the gut and kidney. Moreover, STC‐1 is expressed in vascularized tissue such as heart, kidney, and lung, and is implicated in vascular endothelial cell activation.^6^, ^7^, ^8^ Although STC‐1 plays important roles in host physiology and health, STC‐1‐null mice do not exhibit abnormalities in appearances, development, fertility, and life span.^9^ In addition to the known STC‐1 roles, recent studies have reported that STC‐1 confers growth and anti‐apoptotic properties in lung cancer cells, enhances glucose consumption, ATP production, and lactate production under normoxic and hypoxic conditions, which are key malignancy characteristics, that is, Warburg effects.^10^ Furthermore, some leukemia cell lines express STC‐1, and it has been shown that STC‐1 mRNA levels in peripheral blood correlate with the risk of disease recurrence after chemotherapy.^11^ These reports suggest that suicide gene therapy targeting STC‐1‐expressing cells is a promising strategy for lung cancer treatment.

In this study, we first confirmed STC‐1 expression in plasma samples and tissue slides from lung cancer patients. Next, we tested suicide gene therapy feasibility using a vector expressing uracil phosphoribosyltransferase (UPRT) driven by the STC‐1 promoter in combination with 5‐fluorouracil (5‐FU) treatment in vitro and in vivo.

## 2 METHODS

### 2.1 Cell lines

Human lung cancer cell lines LC‐1/Sq (RCB0455), LC‐2/Ad (RCB0440), LK‐2 (RCB1970), and PC‐9 (RCB4455) were obtained from Riken Bio Resource Research Center (Riken BRC) (Tsukuba, Japan), and A549 (CCL‐185), H1299 (CRL‐5803), H1975 (CRL‐5908) and H1650 (CRL‐5883) were obtained from American Type Culture Collection (ATCC) (Manassas, VA). LC‐1/Sq and LC‐2/Ad were cultured in medium consisting of a mixture of Ham's F12 (N6658) (Sigma‐Aldrich Corp.) and RPMI‐1640 (R8758) (Sigma‐Aldrich Corp.) at a 1:1 (v/v) ratio supplemented with 25 mM HEPES (15630106) (Thermo Fisher Scientific Inc., Waltham, MA), 10% (v/v) FBS (S0250) (Biowest SAS, France) and 100 Units/ml of penicillin and 100 µg/ml of streptomycin (15140122) (Thermo Fisher Scientific Inc.). Also, LK‐2, PC‐9 and H1650 were cultured in RPMI‐1640 (Sigma‐Aldrich Corp.) supplemented with 10% (v/v) FBS (Biowest SAS) and 100 Units/ml of penicillin and 100 µg/ml of streptomycin (Thermo Fisher Scientific Inc.). A549, H1299 and H1975 were cultured in DMEM (D5796) (Sigma‐Aldrich Corp.) supplemented with 10% (v/v) FBS (174012) (Nichirei Corp.) and 100 Units/ml of penicillin and 100 µg/ml of streptomycin (Thermo Fisher Scientific Inc.). Human leukemia cell line K562 (CCL‐243) was obtained from ATCC and cultured in RPMI‐1640 (Sigma‐Aldrich Corp.) supplemented with 10% (v/v) FBS (Biowest SAS) and 100 Units/ml of penicillin and 100 µg/ml of streptomycin (Thermo Fisher Scientific Inc.). HEK293gp cell line (RCB2354) was obtained Riken BRC and cultured in RPMI‐1640 (Sigma‐Aldrich Corp.) supplemented with 10% (v/v) FBS (Biowest SAS). All cells were cultured at 37°C in a humidified incubator containing 5% CO_2_ in air.

### 2.2 RT‐PCR

For reverse transcription‐polymerase chain reaction (RT‐PCR), total cellular RNA was extracted from lung cancer cell lines and collected tumor masses, respectively, using an RNeasy Plus Mini kit (74134) (Qiagen Inc.) and Sepasol‐RNA I Super G (09379–26) (Nacalai Tesque Inc.). Two micrograms of total RNA was reverse‐transcribed to cDNA using ReverTra Ace (TRT‐101) (Toyobo Co. Ltd.) combined with oligo(dT) primers annealed to 5´ ends of the poly(A) tails at 50°C according to the manufacturer's instructions. The generated cDNA was amplified by PCR using GeneAmp PCR System 9700 thermal cycler (Thermo Fisher Scientific Inc.) and Takara ExTaq DNA polymerase (RR001A) (Takara Bio Inc., Japan) for 35 cycles at 95°C for 30 s, 56°C for 30 s and 72°C for 30 s. The products were resolved on a 2% agarose gel and detected by SYBR Green staining. The housekeeping gene β‐actin was served as a control for cDNA synthesis. The following primer pairs were used for this study: Human stanniocalcin‐1 (STC‐1; Accession number NM_003155), 5´‐TTCGGATCCATGCTCCAAAACTCAG‐3´ and 5´‐TTCACGCGTTTATGCACTCTCATGG‐3´; Uracil Phosphoribosyltransferase (UPRT; Accession number X57104), TTGCGGCCGCCACCATGAAGATCGTGGAAGTCAAACACC‐3´ and 5´‐TTCTTAAGTTATTTCGTACCAAAGATTTTGTCACC‐3´; and ACTB (β‐actin; Accession number NM_001101), 5′‐GTGGGGCGCCCCAGGCACCA‐3′ and 5′‐CTCCTTAATGTCACGCACGATTTC‐3′.

### 2.3 ELISA

Culture supernatants were collected from the eight lung cancer cell lines. STC‐1 concentrations in the supernatant and the plasma were measured using a Human Stanniocalcin 1 DuoSet ELISA kit (DY2958) (Bio‐Techne, Minneapolis, MN) according to the manufacturer's instructions. STC‐1 concentrations in culture supernatants were normalized with protein amount in each sample, and the same experiments were repeated three times with different samples. Absorbance at 450 nm was read using SpectraMax M2 microplate reader (Molecular Devices, LLC.).

### 2.4 Construction of reporter plasmids

For construction of reporter plasmids, genomic DNA from human leukemia cell line K562 was used. The fragments of human STC‐1 promoter region were amplified by PCR using KOD ‐Plus‐ Neo (KOD‐401) (Toyobo Co. Ltd.) DNA polymerase and genomic DNA extracted from K562 as a template. PCR was conducted using GeneAmp PCR System 9700 thermal cycler (Thermo Fisher Scientific Inc.) for 35 cycles at 98°C for 10 s, 56°C for 30 s, and 68°C for 60 seconds. The PCR products for each primer pair were digested with *Xho*I/*Bgl*II and were then subcloned into the *Xho*I/*Bgl*II sites of pGL4.10[*luc2*] vector (9PIE665) (Promega Corp.). The following forward primers were paired with the common reverse primer in this study:

Forward primers: for the −931 construct, 5´‐AACTCGAGCTGGGCTTGAACACAGATTATTA‐3´; for the −570 construct, 5´‐AACTCGAGAAGGGCTCAAATGTGTGAGTGAGA‐3´; for the −114 construct, 5´‐AACTCGAGAATTGCATGCCCTCTTATTGGCT‐3´; for the +1 construct, 5´‐AACTCGAGTTTGCAAAAGCCAGAGGTG‐3´; for the +121 construct, 5´‐AACTCGAGCAGCATCACCAGCAACAA‐3´. Reverse primer: 5´‐TTAGATCTGAGAAGTTTCCGCTAAGTTGTTG‐3´.

Reporter plasmids in which NFIC‐ or GATA2‐binding motifs were deleted were generated by inverse PCR method using KOD ‐Plus‐ Neo (Toyobo Co. Ltd.) DNA polymerase and pGL4.10[*luc2*] containing −114 construct as a template. PCR was conducted using GeneAmp PCR System 9700 thermal cycler (Thermo Fisher Scientific Inc.) for 35 cycles at 98°C for 10 seconds, 56°C for 30 seconds, and 68°C for 60 seconds. The PCR products were self‐ligated and were subjected to gene sequence analysis for confirmation of the sequence. The −114 construct lacking motifs for NFIC‐binding site or GATA2‐binding site were subcloned into the *Xho*I/*Bgl*II sites of pGL4.10[*luc2*] vector (Promega Corp.). The following primer pairs with a phosphate group at the 5´ end were used for this study: for deletion of NFIC‐binding motif, 5´‐TAAGAGGGCATGCAATTCAGC‐3´ and 5´‐CACCAGACCAGTTGAGGGAC‐3´; for deletion of GATA2‐binding motif, 5′‐TCCTCAGGATCAAGGACCAA‐3′ and 5´‐CGGCTATAAAATCCCTGGGT‐3´.

### 2.5 Luciferase reporter assay

PC‐9, A549, and H1299 lung cancer cells were seeded in a 96‐well plate at 1.0 × 10^4^ cells and transfected with 100 ng luciferase reporter plasmid and 100 ng of pGL4.74[*hRluc*/TK] (9PIE692) (Promega Corp.). The transfected cells were incubated at 37ºC for 24 h and then assayed using Dual Glo Luciferase Assay System (E2920) (Promega Corp.) according to the manufacturer's instruction, and light intensity was detected by a GloMax Discover Multiplate Reader GM3000 (Promega Corp.). Reporter assays were repeated independently three times with different samples. The relative light intensity was calculated using the following formula.

Relative light intensity (RLT) = [light intensity (Firefly)] / [light intensity (*Renilla*)].

### 2.6 Construction of suicide vector

A vector that expresses a suicide gene UPRT under the control of a STC‐1 promoter was established. Plasmid pHMCMV9 was kindly provided by Dr. Hiroyuki Mizuguchi (Osaka University Graduate School of Pharmaceutics, Osaka, Japan) and was used as a backbone vector.^12^ The −114 construct identified in this study was used as an STC‐1 promoter. The −114 construct was amplified by PCR using KOD ‐Plus‐ Neo (Toyobo Co. Ltd.) DNA polymerase, the reporter plasmid used for this study as a template. The primer sets and conditions of PCR were same as described in above. Plasmid pHMCMV9 was digested with *Apa*I. Then CMV promoter was removed from the backbone vector. Instead, after the sticky ends of the vector were blunted with T4 DNA polymerase (2040A) (Takara Bio Inc.), the −114 construct was inserted as a STC‐1 promoter (pHMCMV9‐*P*_STC‐1_). A UPRT gene was amplified by PCR using KOD ‐Plus‐ Neo (Toyobo Co. Ltd.) DNA polymerase and genomic DNA from *E*. *coli* DH5α (9057) (Takara Bio Inc.) as a template. PCR was conducted using GeneAmp PCR System 9700 thermal cycler (Thermo Fisher Scientific Inc.) for 35 cycles at 98°C for 10 s, 56°C for 30 s, and 68°C for 60 s. The PCR product was digested with *Not*I/*Afl*II and was then subcloned into the *Not*I/*Afl*II sites of pHMCMV9‐*P*_STC‐1_ (pHMCMV9‐*P*_STC‐1_‐UPRT). Next, an IRES‐EGFP cassette was inserted downstream of the *P*_STC‐1_‐UPRT cassette as a marker for cell sorting. An IRES‐EGFP cassette was amplified by PCR using KOD ‐Plus‐ Neo (Toyobo Co. Ltd.) DNA polymerase and plasmid pIRES2‐EGFP (Takara Bio Inc.) as a template. PCR was conducted using GeneAmp PCR System 9700 thermal cycler (Thermo Fisher Scientific Inc.) for 35 cycles at 98°C for 10 s, 56°C for 30 s, and 68°C for 60 s. The PCR product was digested with *Afl*II and was then subcloned into the *Afl*II sites of pHMCMV9‐*P*_STC‐1_‐UPRT (pHMCMV9‐*P*_STC‐1_‐UPRT‐IRES‐EGFP). A vector lacking a UPRT gene was also prepared as a control (pHMCMV9‐*P*_STC‐1_‐Null‐IRES‐EGFP). The following primer pairs were used for this study: −114 construct (a phosphate group was added at the 5´ end), 5´‐TTGGGCCCGAATTGCATGCCCTCTTATTGGCT‐3´ and 5´‐ AACTTAAGTTGCGGCCGCTGAGAAGTTTCCGCTAAGTTGTTG‐3´; UPRT, 5′‐TTGCGGCCGCCACCATGAAGATCGTGGAAGTCAAACACC‐3′ and 5´‐TTCTTAAGTTATTTCGTACCAAAGATTTTGTCACC‐3´; IRES‐EGFP, 5′‐AACTTAAGAATTCTGCAGTCGACGGTACCGCGG‐3′ and 5´‐TTCTTAAGTTACTTGTACAGCTCGTCCATGCCG‐3´.

### 2.7 Establishment of lung cancer cell lines stably expressing a UPRT gene

Next, lung cancer cell lines that stably express UPRT gene under the control of a STC‐1 promoter were established. One million of three lung cancer cell lines confirmed to express STC‐1 (PC‐9, A549, and H1299) were transfected with 1.0 µg of pHMCMV9‐*P*_STC‐1_‐UPRT‐IRES‐EGFP or pHMCMV9‐*P*_STC‐1_‐Null‐IRES‐EGFP and 0.1 µg of pRetroX‐Tight‐Pur (632105) (Takara Bio Inc.) for expression of puromycin‐resistant gene using Cell Line Nucleofector Kit T (Lonza Group Ltd.) and 4D‐Nucleofector (Lonza Group Ltd.) according to the manufacturer's instructions. After transfection, all cells were collected, cultured at 37°C for 24 h, and thereafter subjected to drug selection of 2.0 µg/ml of puromycin (A1113803) (Thermo Fisher Scientific Inc.). The culture media were changed every 3 days. After 2 weeks, the surviving cells were collected and stable transfectants were sorted based on EGFP expression. Briefly, a population of living cells was gated with FSC (x‐axis) versus SSC (Side scatter) (y‐axis) profile (both in linear scale). To obtain single cell population, doublets were gated out with FSC‐H (x‐axis) versus FSC‐W (y‐axis) and SSC‐H (x‐axis) vs. SSC‐W (y‐axis) profiles (all in linear scale). A population that stably expresses *P*_STC‐1_‐UPRT‐IRES‐EGFP (*P*_STC‐1_‐UPRT) or *P*_STC‐1_‐IRES‐EGFP (*P*_STC‐1_‐Null) was gated with a higher EGFP intensity. The EGFP (x‐axis) and the SSC (y‐axis) are in logarithmic scale and linear scale respectively. Then EGFP‐positive cells (i.e., stable transfectants) were sorted using BD FACSAriaII (BD Biosciences). Three lung cancer cell lines that express UPRT (*P*_STC‐1_‐UPRT‐PC‐9/A549/H1299) were used for additional experiments. Those lacking UPRT (*P*_STC‐1_‐Null‐PC‐9/A549/H1299) were used as controls for each cell.

### 2.8 Establishment of lung cancer cell line stably expressing a luciferase gene

The pQCXIN vector (631514) (Takara Bio Inc.) expressing a luciferase gene (hereinafter pQCXIN‐luc) was kindly provided by Dr. Masahiko Kanehira (University of Yamanashi Center for Life Science Research, Yamanashi, Japan). The firefly luciferase gene (*Photinus pyralis luciferase*; Accession number M15077.1) was amplified by PCR using plasmid pGL4.10 [*luc2*] (9PIE665) (Promega) as a template. PCR was conducted using GeneAmp PCR System 9700 thermal cycler (Thermo Fisher Scientific Inc.) and KOD ‐Plus‐ Neo (Toyobo Co. Ltd.) DNA polymerase for 35 cycles at 98°C for 10 s, 60°C for 30 s, and 68°C for 60 s. Primer pair which creates *Not*I and *EcoR*I restriction sites: forward 5´‐AAGCGGCCGCCATGGAAGACGCCAAAAACAT‐3´, reverse, 5´‐TTGAATTCTTACACGGCGATCTTGCCGC‐3´. The obtained fragment was subcloned into the *Not*I/*EcoR*I site of pQCXIN vector (Figure. S1B). HEK293gp cells were used for production of retroviral vector. Two micrograms of the pQCXIN‐luc was co‐transfected with 0.2 µg of pVSV‐G vector (631530) (Takara Bio Inc.) into HEK293gp cells using Lipofectamine 2000 (11668027) (Thermo Fisher Scientific Inc.) according to the manufacturer's instructions. After 48 h, culture supernatants containing retrovirus particles were collected, passed through a 0.45‐µm filter and used for infection of LK‐2. The medium was changed 24 h later. Drug selection with 400 µg/ml of G‐418 (4727878001) (Roche, Switzerland) was started 72 h later. The medium was changed every 3 days. After 2 weeks, the surviving cells (i.e., stable transfectant) were then expanded in G‐418 containing medium and were used for additional experiments.

### 2.9 Proliferation assay

Three lung cancer cell lines, *P*_STC‐1_‐UPRT‐PC‐9/A549/H1299, or their controls, were seeded at a density of 5,000 cells/0.2 ml into a 96‐well plate and were cultured at 37°C. After 24 h, cells were treated with 0−50 µM of 5‐fluorouracil (5‐FU) (F6627) (Sigma‐Aldrich Corp.) for 72 h. The arrest of cell growth was evaluated using CellTiter 96 AQueous Non‐Radioactive Cell Proliferation Assay (G5421) (Promega Corp.) according to the manufacturer's instructions. Experiments were repeated independently three times with triplicate samples. The absorbance at 490 nm was read using SpectraMax M2 microplate reader (Molecular Devices, LLC.). The cell growth percentage was calculated using the following formula.

Cell growth (%) = (mean absorbance [treated well] – mean absorbance [blank])/(mean absorbance [untreated well] – mean absorbance [blank]) ×100.

Similarly, 2.5 × 10^3^ cells of *P*_STC‐1_‐UPRT‐PC‐9/A549/H1299 were seeded into a well of 96‐well plate and were cultured at 37°C. After 24 h, cell proliferation was stopped with 10 µg/ml of Mitomycin C (M3377) (Fujifilm Wako Pure Chemical Corp.). Then 2.5 × 10^3^ of LK‐2 was added (total 5 × 10^3^ cells/well) to a well in which *P*_STC‐1_‐UPRT‐PC‐9/A549/H1299 were seeded, or 5 × 10^3^ of LK‐2 was added to an empty well as a control. Cells were treated with 0−25 µM of 5‐FU (Sigma‐Aldrich Corp.) for 72 h. Experiments were repeated independently three times with triplicate samples. Then the arrest of cell growth was evaluated using the method described above.

### 2.10 Tumor xenograft transplantation

Procedures were performed using 4‐week‐old female athymic BALB/c‐nu/nu mice. All mice were purchased from CLEA Japan Inc. (https://www.clea‐japan.com/en/products/immunodeficiency/item_a0010) (Tokyo, Japan) and were kept under sterile conditions. Mice were housed in cages containing compressed paper tip bedding under controlled conditions of temperature (22–24°C), humidity (about 60%), and light (12 h dark/light cycle). All procedures involving animals, including housing and care, methods of euthanasia, and experimental protocols, were performed according to protocols approved by Tohoku University's Institutional Committee for the Use and Care of Laboratory Animals. To establish a tumor xenograft model, 2 × 10^6^ cells of *P*_STC‐1_‐UPRT‐PC‐9 or *P*_STC‐1_‐Null‐PC‐9 were injected subcutaneously into the left flank of mice under anesthesia with a mixture of medetomidine, midazolam, and butorphanol. After 5 days and palpable tumor formation, 5‐FU (Sigma‐Aldrich Corp.) was administered intraperitoneally every other day at a dose of 1.0 mg/kg of body weight. Tumor growth was measured every 2 days using calipers. Then the tumor volume was calculated using the following formula.

Tumor volume (mm^3^) = 0.5 × (length [mm]) × (width [mm])^2^.

All mice were euthanized after 28 days, and the tumor masses were then collected and weighed. These experiments were repeated independently three times using 6 mice per group per experiments. Similarly, 1 × 10^6^ LK‐2 lung tumor cells expressing a luciferase gene (LK‐2‐Luc) were mixed with the same number of *P*_STC‐1_‐UPRT‐PC‐9 or *P*_STC‐1_‐Null‐PC‐9 and were injected subcutaneously into the left flank of 4‐week‐old female BALB/c‐nu/nu mice under anesthesia with a mixture of medetomidine, midazolam, and butorphanol. After 5 days and palpable tumor formation, 5‐FU (Sigma‐Aldrich Corp.) was administered intraperitoneally every other day at a dose of 1.0 mg/kg of body weight. Tumor growth was measured every 2 days using the method described above. All mice were euthanized after 28 days, and the tumor masses were then collected and weighed. These experiment were also repeated independently three times using 3 to 6 mice per group per experiments.

### 2.11 Bioluminescent imaging

To monitor tumor growth noninvasively, mice bearing LK‐2‐Luc were administered intraperitoneally with 150 mg/kg sterile D‐Luciferin (122799) (PerkinElmer Inc.) dissolved in PBS. At 10 min after substrate injection, the mice were anesthetized using 2% isoflurane (0026675–46–7) (MSD Animal Health) and were imaged for 1 s at the maximal light collection rate and the highest resolution using IVIS Lumina II (PerkinElmer Inc.).

### 2.12 Histopathological assay

Tumor‐bearing mice were euthanized with isoflurane (MSD Animal Health). The tumor masses were collected. Tumor masses were fixed at 4°C for 24 h in 4% paraformaldehyde (163–20145) (Fujifilm Wako Pure Chemical Corp.), were embedded in paraffin blocks, and were sectioned at 5 µm thickness using a microtome to prepare tissue slides. After deparaffinized and rehydration, tissue slides were stained with hematoxylin and eosin (H&E) or were immunostained. For immunostaining, the slides were heated for 10 min at 95 to 100°C in 10 mM sodium citrate buffer (pH 6.0) to recover antigenicity and blocked with blocking solution consisting of 0.05% (v/v) Tween 20 (P1379) (Sigma‐Aldrich Corp.) in phosphate buffered saline (PBS‐T) supplemented with 2% (w/v) bovine serum albumin (A5611) (Sigma‐Aldrich Corp.) for 1 h at 20°C. Thereafter, sections were reacted with an anti‐Ki‐67 antibody (LS‐B6433) (LifeSpan BioSciences Inc.) at a dilution of 1:200 in blocking solution for 24 h r at 4°C. After washing with PBS‐T, the sections were reacted with an anti‐Mouse IgG (H + L), HRP Conjugate (W4021) (Promega Corp.) at a dilution of 1:2000 in blocking solution for 1 h at 20°C. After washing with PBS‐T, Ki‐67‐positive nuclei were visualized with DAB chromogen substrate (K3468) (Agilent Technologies Inc.). To detect expression of green fluorescent protein (GFP) in tumor tissue, the collected tumor masses were fixed in 4% paraformaldehyde (Fujifilm Wako Pure Chemical Corp.), embedded in OCT compound (4583) (Sakura Finetechnical Co. Ltd.), and frozen at −80°C. After cryosections were prepared from the frozen blocks at 20 µm thickness using a cryostat (Leica CM1950; Leica Biosystems), nuclei were counterstained with mounting medium with DAPI (4′,6‐diamidino‐2‐phenylindole) (H‐1200) (Vectashield; Vector Laboratories, Burlingame, CA) and were observed using BZ‐X fluorescence microscopy (Keyence Co.). Paraffin‐embedded lung sections from two lung cancer patients, diagnosed as squamous cell carcinoma or adenocarcinoma, were kindly provided by Tohoku University Hospital (Sendai, Japan). The specimens were sectioned at 5 µm thickness and were stained with H&E or were immunostained as described above. Briefly, for detection of STC‐1, sections were reacted with an anti‐Stanniocalcin‐1 Rabbit‐Poly (GTX129092) (Genetex Inc.) at dilution of 1:200 in blocking solution for 24 h at 4°C. After washing with PBS‐T, the sections were reacted with a goat anti‐rabbit Ig FITC conjugated (NB7182) (Bio‐Techne) at a dilution of 1:2000 in blocking solution for 1 h at 20°C. After immunofluorescent staining, nuclei were counterstained with Vectashield mounting medium with DAPI (Vector Laboratories) and were observed using BZ‐X fluorescence microscopy (Keyence Co.). This study was approved by the Institutional Review Board of Tohoku University Graduate School of Medicine (Sendai, Japan).

### 2.13 Determination of STC‐1 levels in blood

Plasma samples were obtained from 20 patients with lung cancer and from 12 healthy volunteers. STC‐1 levels in plasma were found using a Human Stanniocalcin 1 DuoSet ELISA kit (Bio‐Techne) according to the manufacturer's instructions. The Institutional Review Board at Tohoku University Graduate School of Medicine (Sendai, Japan) approved this study.

### 2.14 Statistical analysis

All data are presented as mean and S.D. of the indicated number of samples and replicates. Statistical analyses were carried out using either Microsoft Excel (Microsoft Corp, Redmond, WA, USA) or Statcel 4 software (OMS Publishing Inc.). Significance was inferred from results of Mann–Whitney *U*‐test or Kruskal–Wallis test followed by Steel–Dwass test. *p*‐values of <0.05 were inferred as significant.

## 3 RESULTS

### 3.1 STC‐1 levels in blood samples from lung cancer patients and healthy donors

**FIGURE 1:**
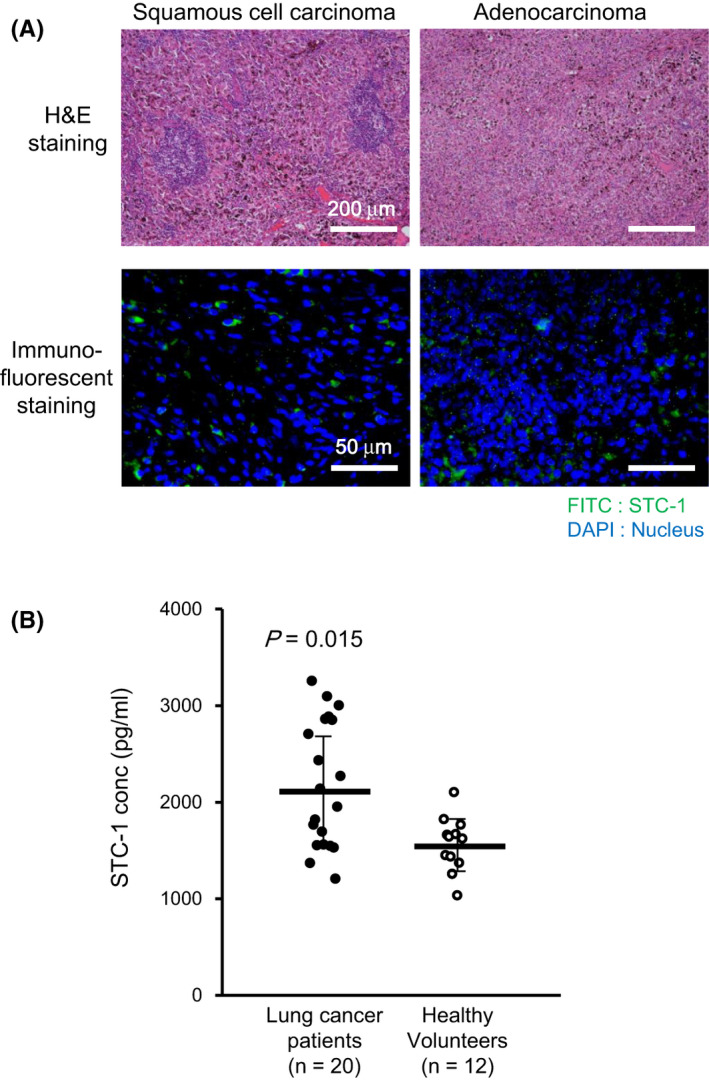
Evaluation of STC‐1 as a novel prognostic and molecular marker for lung cancer. (A) Expression of STC‐1 in lung cancer tissue specimens. Representative images of H&E staining (upper) and immunofluorescence staining for STC‐1 (lower) are shown in adenocarcinoma (left) and squamous cell carcinoma (left). Scale bar (upper): 200 µm, Scale bar (lower): 50 µm. (B) Blood samples were collected from 20 lung cancer patients and 12 healthy volunteers. Serum levels of STC‐1 in lung cancer patients (filled circles) and healthy volunteers (open circles) were determined by ELISA as described in *Materials and Methods*. All results represent means ±S.D. values. *p*‐value was the result of Mann–Whitney *U*‐test

First, we sought to clarify the usefulness of STC‐1 as a molecular target for lung cancer therapy. We confirmed the expression of STC‐1 in specimens from patients diagnosed as having lung cancer, especially squamous cell carcinoma and adenocarcinoma. As presented in Figure 1A, results of immunohistochemical analyses show that many STC‐1‐positive cells were detected in both specimens. Additionally, we measured the STC‐1 levels in blood samples from 20 lung cancer patients and 12 healthy volunteers. It is noteworthy that STC‐1 concentration in plasma was significantly higher in lung cancer patients than in healthy volunteers (Figure 1B). These results suggest STC‐1 as a candidate targeting molecule for lung cancer therapy.

### 3.2 STC‐1 expression in some lung cancer cell lines

**FIGURE 2:**
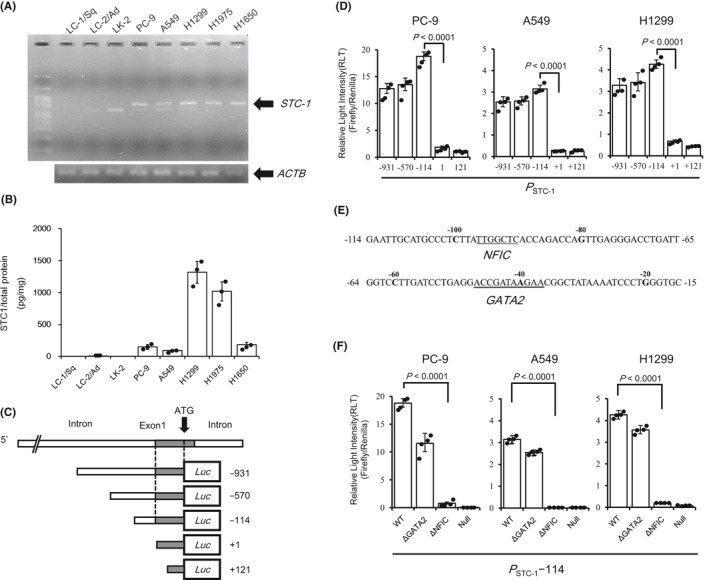
Expression pattern of STC‐1 in various lung cancer cell lines and determination of the promoter region of STC‐1. (A) RT‐PCR analysis for detection of STC‐1 mRNA in eight lung cancer cell lines. STC‐1 mRNA was detected in PC‐9, A549, H1299, H1975, and H1650 cell lines. The bands of β‐actin served as control. (B) ELISA for detection of STC‐1 protein in culture supernatant collected from eight lung cancer cell lines. The bar graph and overlaid dots represent the means ±S.D. values and measured values, respectively, from three independent experiments with different samples. STC‐1 protein was detected in PC‐9, A549, H1299, H1975, and H1650 cell lines in accordance with RT‐PCR results. (C) Schematic representation of STC‐1 promoter‐luciferase reporter plasmids: pGL4.10[*luc2*] vector containing 931 bp, 570 bp, and 114 bp upstream (−931, −570, and −114, respectively), and 1 bp and 121 bp downstream (+1 and +121, respectively) of the first exon of STC‐1. (D) Luciferase assay for examination of promoter activity. PC‐9, A549 and H1299 cells were cotransfected with 100 ng of the STC‐1 promoter (*P*_STC‐1_)‐luciferase construct (−931, −570, −114, +1 or +121) and 100 ng of pGL4.74[*hRluc*/TK]. The bar graph and overlaid dots represent the means ±S.D. values and measured values respectively. This experiment was repeated independently in quadruplicate three times with reproducible result, and a representative result is shown. The light intensity of Firefly was normalized with light intensity of Renilla, and indicated as the relative light intensity (RLT). Significance was inferred from results of Student's two‐tailed *t*‐tests. (E) Nucleotide sequence of the 5’‐flanking of the STC‐1 gene (from −114 to −15). Putative binding sites of transcription factor are underlined. Deduced transcription factors are presented below. (F) Luciferase assay for determination of site‐deleted promoter activity. PC‐9, A549, and H1299 cells were cotransfected with 100 ng of the *P*_STC‐1_‐luciferase construct −114 (WT) and −114 lacking GATA2 or NFIC‐binding site (ΔGATA2 of ΔNFIC, respectively) and 100 ng of pGL4.74[*hRluc*/TK]. The bar graph and overlaid dots represent the means ±S.D. values and measured values respectively. This experiment was repeated independently in quadruplicate three times with reproducible result, and a representative result is shown. The light intensity of Firefly was normalized with light intensity of Renilla and shown as the relative light intensity (RLT). *p*‐values were the results of Kruskal–Wallis test followed by Steel–Dwass test

Second, we monitored expression of STC‐1 in eight lung cancer cell lines. Of the eight lung cancer cell lines, STC‐1 expression was confirmed in five for both mRNA and protein levels. Results show that STC‐1 expression was high in H1299 and H1975, but slight or non‐existent in LC‐1/Sq, LC‐2/Ad, and LK‐2 (Figure 2A and B). Next, we attempted to ascertain the promoter region related to transcription of STC‐1 mRNA using 3 of STC‐1‐expressing lung tumor cell lines. We constructed reporter vectors containing 931 bp, 570 bp, and 114 bp upstream (−931, −570, and −114, respectively), and 1 bp and 121 bp downstream (+1 and +121, respectively) of the first exon of STC‐1 and examined the promoter activity of these regions using reporter assay (Figure 2C). Although promoter activity was detected in −931, −570, and −114, neither +1 nor +121 exhibited any promoter activity (Figure 2D) in all three cell lines. This result suggests that the promoter region of STC‐1 gene might exist within 114 bp upstream of the first exon. Next, we attempted to identify transcription factor(s) responsible for STC‐1 gene transcription. Using the database JASPAR (http://jaspar.genereg.net/) to elucidate the transcription factors that bind to −114, NFIC and GATA2 were identified as candidates (Figure 2E). Then, we evaluated the promoter activity respectively in −114 lacking the binding site of NFIC or GATA2, or of −114 ΔNFIC or −114 ΔGATA2. All of PC‐9, A549, and H1299 showed a marked decrease in promoter activity in −114 ΔNFIC, but not −114 ΔGATA2 (Figure 2F). From these results, NFIC can be inferred as a transcription factor responsible for the transcription of STC‐1.

### 3.3 Establishment of lung cancer cell lines expressing a suicide gene under the control of STC‐1 promoter

**FIGURE 3:**
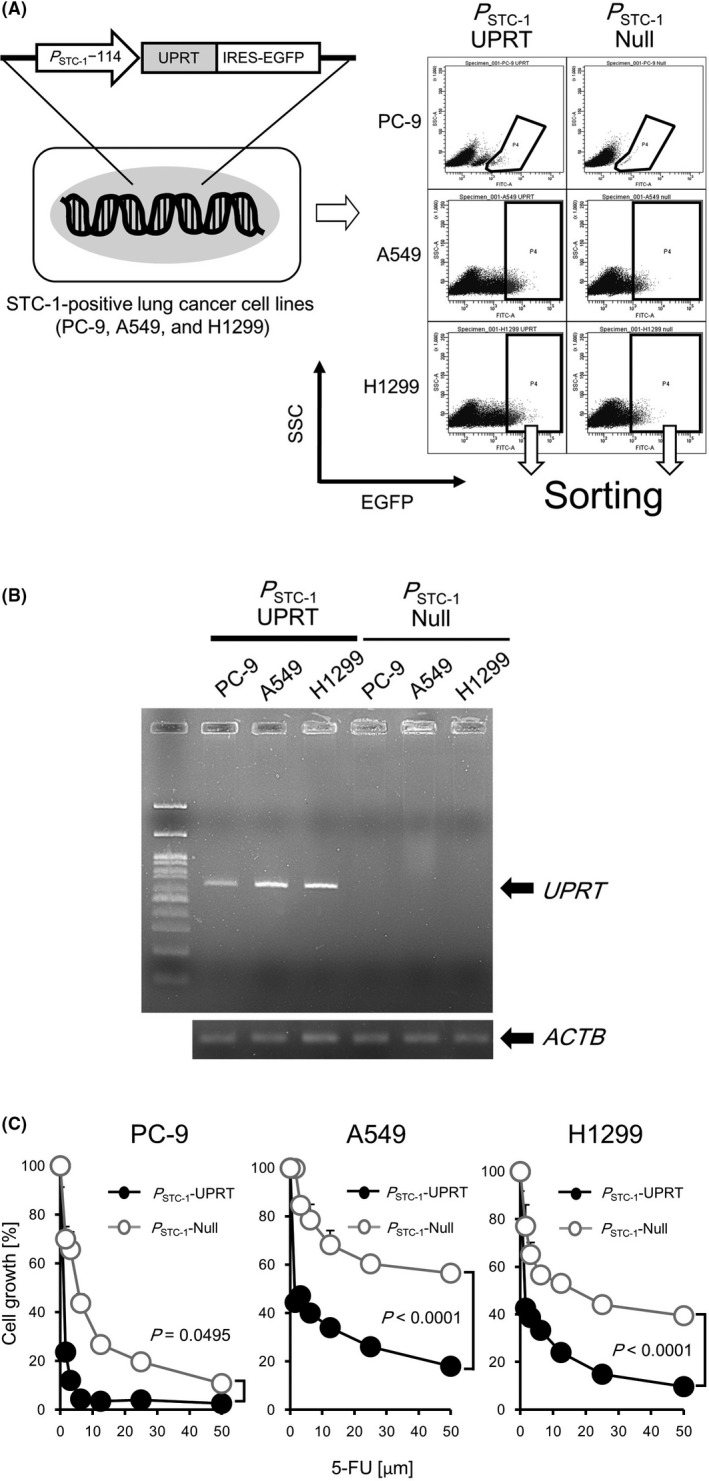
Establishment of cell lines that express suicide gene UPRT under the control of STC‐1‐promoter and evaluation of enhanced growth arrest by 5‐FU in vitro. (A) The structure of an expression cassette for expression of UPRT gene. An IRES‐EGFP sequence was tethered to UPRT gene for cell sorting. An expression cassette lacking UPRT was used as a control (not shown). The expression cassette was transfected to PC‐9, A549, and H1299 cells (left). After selection using puromycin, surviving cells were subjected to cell sorting to collect EGFP‐positive cells (i.e. stable population) and were used for additional experiments (right). The representative histograms are shown. The EGFP (x‐axis) and the SSC (Side scatter) (y‐axis) are shown in logarithmic scale and linear scale respectively. (B) RT‐PCR analysis for detection of UPRT gene in three lung cancer cell lines that were transfected with UPRT‐expressing vector (*P*_STC‐1_‐UPRT‐PC‐9/A549/H1299) and three lung cancer cell lines that were transfected with control vector (*P*_STC‐1_‐Null‐PC‐9/A549/H1299). UPRT mRNA was detected in *P*_STC‐1_‐UPRT‐PC‐9/A549/H1299, but not *P*_STC‐1_‐Null‐PC‐9/A549/H1299. The bands of β‐actin were served as control. (C) Proliferation of *P*_STC‐1_‐UPRT‐PC‐9/A549/H1299 and *P*_STC‐1_‐Null‐PC‐9/A549/H1299 after treatment with 5‐FU (50, 25, 12.5, 6.25, and 3.125, 1,56 and 0 [µM]) were measured by performing MTT assay. All data represent the means ±S.D. values of triplicate samples. This experiment was repeated independently three times with reproducible result, and a representative result is shown. The percentage of cell growth was calculated as described in *Materials and Methods*. *p*‐value was the result of Mann–Whitney *U*‐test. Abbreviations: EGFP, enhanced green fluorescence protein; IRES, internal ribosome entry site; UPRT, uracil phosphoribosyltransferase

To induce antitumor effects specifically to STC‐1‐expressing lung cancer cells, we established lung cancer cell lines of three types that express a suicide gene in an STC‐1 promoter‐dependent manner. We selected 114 bp upstream of the first exon identified in this study as the STC‐1 promoter (*P*_STC‐1_−114). A vector in which the suicide gene uracil phosphoribosyltransferase (UPRT), internal ribosome entry site (IRES), and EGFP gene were ligated downstream of the *P*_STC‐1_–114 was constructed (Figure 3A left and Figure S1A) and transfected into STC‐1‐expressing lung cancer cell lines PC‐9, A549, and H1299. After drug selection, EGFP‐positive (i.e. stable transfectants) cells were sorted using a cell sorter (Figure 3A right and Figure S2). Subsequently, it was confirmed that the three stable transfectants (*P*_STC‐1_‐UPRT‐PC‐9/A549/H1299) all expressed the UPRT gene, but control transfectants (*P*_STC‐1_‐Null‐PC‐9/A549/H1299) did not (Figure 3B). These transfectants were also confirmed to express UPRT gene after several passages in culture (data not shown).

### 3.4 Evaluation of growth‐arresting effects by STC‐1 promoter‐dependent UPRT expression

**FIGURE 4:**
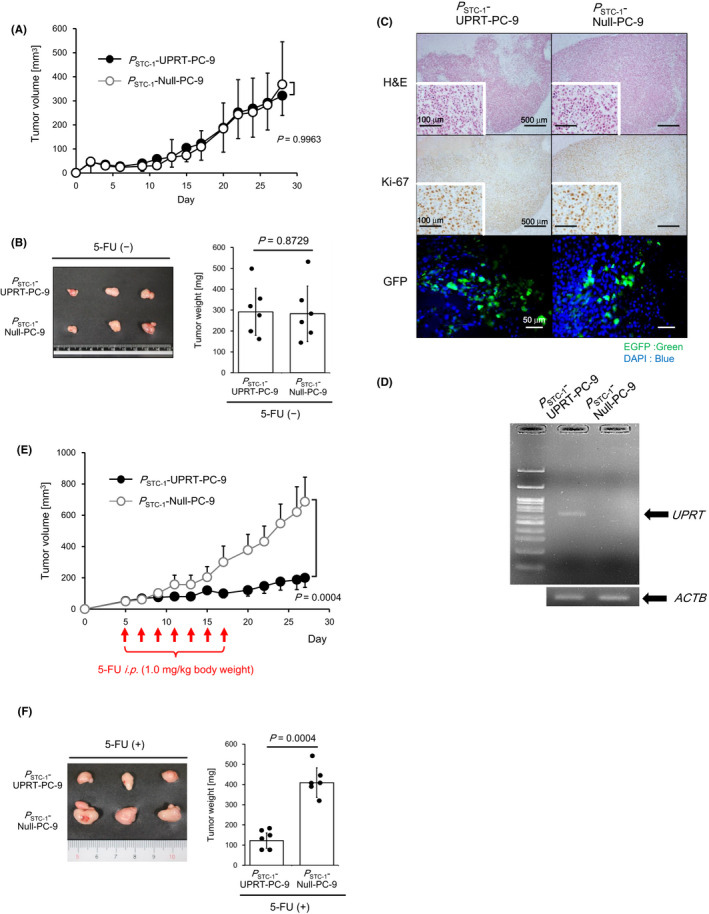
Evaluation of antitumor effects by targeting STC‐1‐expressing lung cancer cells by the UPRT/5‐FU system in vivo. (A) Establishment of a human lung cancer xenograft model using nude mice. The size of the tumor formed by *P*_STC‐1_‐UPRT‐PC‐9 (filled circle) or *P*_STC‐1_‐Null‐PC‐9 (open circle) in mice (*n* = 8 for each group) was measured without 5‐FU treatment. All data represent the means ±S.D. values. This experiment was repeated independently three times with reproducible result, and a representative result is shown. (B) Tumor growth was evaluated without 5‐FU treatment. Macroscopic findings (left) and weight (right) of collected tumors. The bar graph and overlaid dots represent the means ±S.D. values and measured values respectively. This experiment was repeated independently three times with reproducible result, and a representative result is shown. (C) Histopathologic findings of collected tumors formed by *P*_STC‐1_‐UPRT‐PC‐9 or *P*_STC‐1_‐Null‐PC‐9. Representative microscopic images of hematoxylin and eosin staining (upper) and anti‐Ki‐67 immunohistochemistry (middle). Scale bar: 500 µm (scale bar in inset: 100 µm). Representative microscopic images of fluorescence in a tumor section (bottom) Scale bar: 50 µm. (D) RT‐PCR for detection of UPRT mRNA in tumor mass formed by *P*_STC‐1_‐UPRT‐PC‐9 or *P*_STC‐1_‐Null‐PC‐9. Bands of β‐actin served as control. (E) Enhanced antitumor effects by systemic administration of 5‐FU. Every second day from day 5 through day 17 (seven times total), 5‐FU (1.0 mg/kg body weight) was administered intraperitoneally into tumor‐bearing mice. The tumor size of *P*_STC‐1_‐UPRT‐PC‐9 (filled circle) or *P*_STC‐1_‐Null‐PC‐9 (open circle) in mice (*n* = 8 for each group) was measured. All data represent the means ±S.D. values. This experiment was repeated independently three times with reproducible result, and a representative result is shown. (F) Tumor growth was evaluated after 5‐FU treatment. Macroscopic findings (left) and weight (right) of collected tumors. The bar graph and overlaid dots represent the means ±S.D. values and measured values respectively. This experiment was repeated independently three times with reproducible result, and a representative result is shown. *p*‐values were the results of Mann–Whitney *U*‐test

First, to evaluate whether a sufficient growth‐arresting effect is obtainable by expressing UPRT, the three cell lines of *P*_STC‐1_‐UPRT‐PC‐9/A549/H1299 or their controls were treated with 5‐fluorouracil (5‐FU). Thereafter, cell growth was monitored. As expected, the growth of each of *P*_STC‐1_‐UPRT‐PC‐9/A549/H1299 was significantly arrested compared to their respective *P*_STC‐1_‐Null‐PC‐9/A549/H1299 controls in a dose‐dependent manner of 5‐FU (Figure 3C). For the next experiment, we further confirmed the growth‐arresting effects accomplished by 5‐FU administration using a mouse xenograft model. Before the experiments, *P*_STC‐1_‐UPRT‐PC‐9 or *P*_STC‐1_‐Null‐PC‐9 was inoculated subcutaneously into BALB/c‐nu/nu mice. Their capability to form a subcutaneous tumor mass was confirmed. Both *P*_STC‐1_‐UPRT‐PC‐9 and *P*_STC‐1_‐Null‐PC‐9 formed visible tumor masses up to 30 days after inoculation. No difference was found in terms of growth rate, volume, or weight between these two cell lines (Figure 4A and B). In accordance with the results, no difference in pathological findings was obtained between tumor masses of *P*_STC‐1_‐UPRT‐PC‐9 and *P*_STC‐1_‐Null‐PC‐9, such as invasion of cells with dense nuclear staining and positive for Ki‐67, and rich stroma in tumor tissues (Figure 4C, upper and middle). In addition, EGFP‐positive cells were detected in both tumor tissues (Figure 4C, lower). The UPRT gene was detected in tumor tissues of *P*_STC‐1_‐UPRT‐PC‐9 but not of *P*_STC‐1_‐Null‐PC‐9 (Figure 4D). These results imply that the STC‐1 promoter we used for this study is sufficient to drive gene expression in mouse tissues in which the cells were transplanted. In line with these results, *P*_STC‐1_‐UPRT‐PC‐9 or *P*_STC‐1_‐Null‐PC‐9 was inoculated subcutaneously into BALB/c‐nu/nu mice. Growth‐arresting effects observed in vitro were confirmed in vivo. After palpable tumors were formed, 5‐FU was administered intraperitoneally seven times every second day. Tumor volumes were measured every second day. As shown in Figure 4E, tumor growth of *P*_STC‐1_‐UPRT‐PC‐9 was arrested significantly more than that of *P*_STC‐1_‐Null‐PC‐9. In accordance with the result, at 27 days after inoculation, the tumor mass of *P*_STC‐1_‐UPRT‐PC‐9 was about one‐fourths that of *P*_STC‐1_‐Null‐PC‐9 (Figure 4F). From these results, we inferred that the targeting of STC‐1‐expressing lung cancer cells was sufficient to obtain efficient growth‐arresting effects of tumors.

### 3.5 Evaluation of growth‐arresting effects against adjacent STC‐1‐negative tumor cells

**FIGURE 5:**
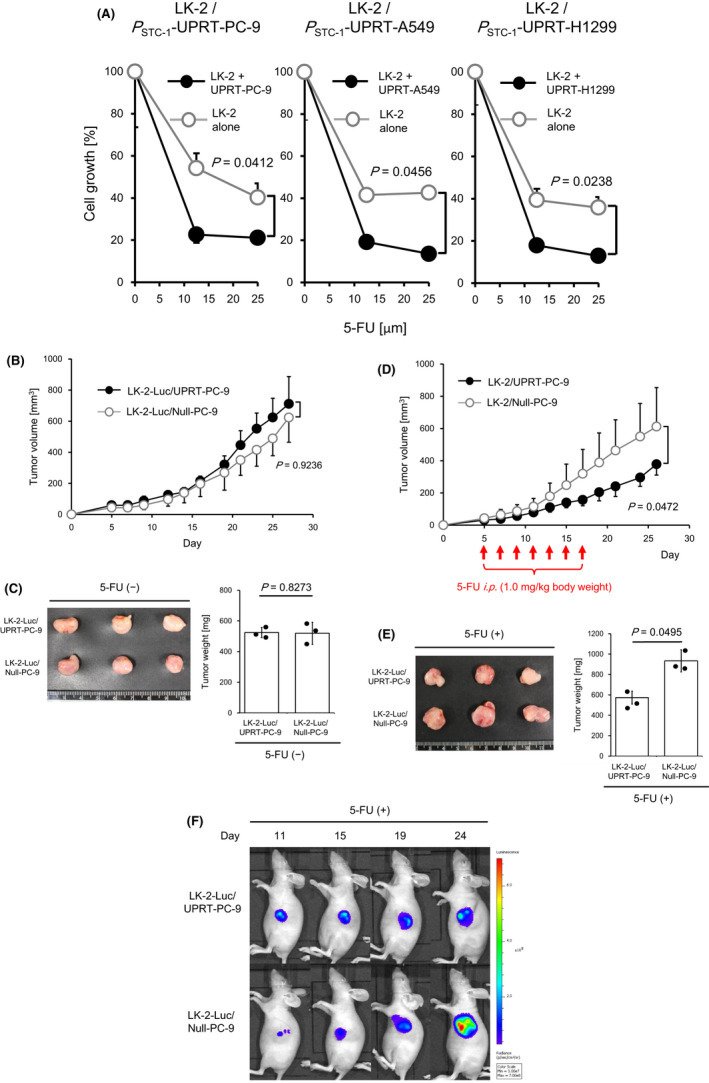
Evaluation of antitumor effects against STC‐1‐negative lung cancer cells by targeting adjacent STC‐1‐expressing lung cancer cells in vitro and in vivo. (A) Proliferation of STC‐1‐negative LK‐2 lung cancer cells with or without *P*_STC‐1_‐UPRT‐PC‐9/A549/H1299 after treatment with 5‐FU (25, 12.5, and 0 [µM]) were measured by performing MTT assay. Proliferation of *P*_STC‐1_‐UPRT‐PC‐9/A549/H1299 was halted by treatment with 10 µg/ml of Mitomycin C. All data represent the means ±S.D. values of triplicate samples. This experiment was repeated independently three times with reproducible result, and a representative result is shown. The percentage of cell growth was calculated as described in *Materials and Methods*. (B) Establishment of a heterogeneous human lung cancer model using nude mice. LK‐2 lung cancer cells that exogenously expressed luciferase gene (LK‐2‐Luc) were mixed with *P*_STC‐1_‐UPRT‐PC‐9 or *P*_STC‐1_‐Null‐PC‐9 and inoculated subcutaneously into the left flank of nude mice. The sizes of tumors formed by LK‐2‐Luc/UPRT‐PC‐9 (filled circle) or LK‐2‐Luc/Null‐PC‐9 (open circle) in mice (*n* = 8 for each group) were measured without 5‐FU treatment. All data represent the means ±S.D. values. This experiment was repeated independently three times with reproducible result, and a representative result is shown. (C) Tumor growth was evaluated without 5‐FU treatment. Macroscopic findings (left) and weight (right) of collected tumors. The bar graph and overlaid dots represent the means ±S.D. values and measured values respectively. This experiment was repeated independently three times with reproducible result, and a representative result is shown. (D) Evaluation of bystander antitumor effects via colocalized *P*_STC‐1_‐UPRT‐PC‐9 and systemic administration of 5‐FU. After palpable tumor formation, every second day from day 5 through day 17 (total seven times), mice were administered 5‐FU (1.0 mg/kg body weight) intraperitoneally. The tumor size of LK‐2‐Luc/UPRT‐PC‐9 (filled circle) or LK‐2‐Luc/Null‐PC‐9 (open circle) in mice (*n* = 8 for each group) was measured. All data represent the means ±S.D. values. This experiment was repeated independently three times with reproducible result, and a representative result is shown. (E) Tumor growth was evaluated after 5‐FU treatment. Macroscopic findings (left) and weight (right) of collected tumors. All results represent the mean and SD of three experiments. The bar graph and overlaid dots represent the means ±S.D. values and measured values, respectively. This experiment was repeated independently three times with reproducible result, and a representative result is shown. (F) Representative images from in vivo bioluminescence assays. Mice transplanted with LK‐2‐Luc/UPRT‐PC‐9 (upper) or LK‐2‐Luc/Null‐PC‐9 (lower) were monitored on days 11, 15, 19, and 24. Results represent those of three independent experiments. *p*‐values were the results of Mann–Whitney *U*‐test

Solid tumors are widely known to consist of heterogeneous populations of tumor cells. We also confirmed that not all lung cancer cells express STC‐1 (Figure 2A and B). Wei et al. reported that 5‐FU and its derivatives readily diffuse into the interstitial space, that they are incorporated into surrounding cells, and that they exert a strong bystander cytotoxic effect.^13^ For the last study, we validated whether targeting of STC‐1‐positive lung cancer cells by 5‐FU can also exert growth‐arresting effects against adjacent STC‐1‐negative lung cancer cells. First, STC‐1‐negetive LK‐2 lung cancer cells were co‐cultured with mitomycin‐treated *P*_STC‐1_‐UPRT‐PC‐9/A549/H1299 and were treated with 5‐FU. Then cell growth was monitored. As shown in Figure 5A, the growth of LK‐2 cells was arrested significantly more by co‐culturing with all *P*_STC‐1_‐UPRT‐PC‐9/A549/H1299 than were LK‐2 cells alone in a dose‐dependent manner of 5‐FU. Next, we further verified growth arrest against LK‐2 cells exerted by adjacent STC‐1‐positive cells using a mouse xenograft model. Before the experiments, luciferase‐expressing LK‐2 cells (hereinafter LK‐2‐Luc) were mixed with *P*_STC‐1_‐UPRT‐PC‐9 or *P*_STC‐1_‐Null‐PC‐9 (LK‐2‐Luc/ UPRT‐PC‐9‐ or LK‐2‐Luc/Null‐PC‐9, respectively) and were inoculated subcutaneously into BALB/c‐nu/nu mice. Their capability to form a subcutaneous tumor mass was confirmed. Both LK‐2‐Luc/UPRT‐PC‐9 and LK‐2‐Luc/Null‐PC‐9 successfully formed visible tumor masses up to 30 days after inoculation. No difference was found in terms of growth rate, volume, or weight between these two groups (Figure 5B and C). Next, LK‐2‐Luc/UPRT‐PC‐9 or LK‐2‐Luc/Null‐PC‐9 were inoculated subcutaneously into BALB/c‐nu/nu mice. After palpable tumors formed, 5‐FU was administered intraperitoneally seven times every second day. Tumor volumes were measured every second day. As shown in Figure 5D and E, the growth of LK‐2‐Luc/UPRT‐PC‐9 was significantly more arrested than in LK‐2‐Luc/Null‐PC‐9 in terms of both weight and volume. Furthermore, we confirmed that the results observed for mice inoculated with LK‐2‐Luc/UPRT‐PC‐9 and treated with 5‐FU were attributed to growth arrest of both LK‐2‐Luc and UPRT‐PC‐9, and not to that of UPRT‐PC‐9 alone. As observed using bioluminescence assay, the mice inoculated with LK‐2‐Luc/UPRT‐PC‐9 exhibited significantly more quenching of luminescence at days 19 and 24 than that exhibited by LK‐2‐Luc/Null‐PC‐9 by administration of 5‐FU (Figure 5F). These results suggest strongly that targeting of STC‐1‐positive tumor cells by expression of UPRT gene is sufficient to exert growth‐arresting effects against adjacent STC‐1‐negative tumor cells.

## 4 DISCUSSION

To date, many anticancer drugs have been developed. Cancer chemotherapy has made great progress. However, acquisition of drug resistance, distant metastasis, and recurrence are observed in many cancer cases. Many studies have emphasized development of new therapeutic agents, and have particularly allocated attention to finding molecular targets that act on cancer‐specific signal transduction to minimize effects on normal cells and to suppress adverse events. This study investigated the possibility of using STC‐1 as a new molecular target for lung cancer. In this study, we confirmed that STC‐1 was detected in tissue specimens and peripheral blood samples from lung cancer patients. It is noteworthy that the STC‐1 level was significantly higher in lung cancer patients than in healthy volunteers. And also, the expression of STC‐1 was observed in tissues of squamous cell carcinoma and adenocarcinoma, both of which are grouped as non‐small cell lung carcinoma (NSCLC). On the other hand, in our results using eight lung cancer cell lines, STC‐1 expression was observed in five. Although cell lines originated from adenocarcinoma (e.g. PC‐9, A549, H1975, and H1650), and large cell carcinoma (e.g. H1299) expressed STC‐1, cell lines originated from squamous cell carcinoma (e.g. LC‐1/Sq and LK‐2) did not. Moreover, the expression levels differed greatly among the five cell lines. These results seem to reflect that cancer tissues are heterogeneous populations composed of various cancer cells, even though collectively grouped as NSCLC. In an earlier report, STC‐1 expression was recognized in 8 of 10 leukemia cell lines. For acute leukemia, a relation between the expression level of STC‐1 mRNA in peripheral blood and recurrence after chemotherapy has been suggested.^11^ In addition to leukemia cells, STC‐1 expression has been found to be increased in various cancers such as colorectal cancer and ovarian cancer.^14^, ^15^, ^16^ These reports suggest that, although STC‐1 is not a molecule found only in lung cancer cells, it might be a molecule that positively regulates the progression of cancers. Therefore, it would be meaningful to evaluate the possibility of STC‐1 further as a molecular target of lung cancer and to clarify whether STC‐1 expression correlates with tumor malignancy and poor prognosis.

We first clarified from this study that Nuclear factor I‐C (NFIC) is a transcription factor responsible for transcription of STC‐1 gene. The NFI family, which consists of four evolutionally conserved members NFIA, NFIB, NFIC, and NFIX, functions as an essential transcription factor for the development of several organs as well as an adenovirus DNA replication factor.^17^ Especially, NFIC is known as an important transcription factor for the differentiation and development of epithelium, bone, and blood cells.^18^ In addition, NFIC is involved in the development of several malignancies including breast cancer, brain tumor, and lung squamous cell carcinoma via the cell cycle and DNA replication pathway.^19^, ^20^, ^21^ We performed the pathway analysis using the STING website (https://string‐db.org/). The result showed that CDC5L (Cell division cycle 5‐like protein) and ECD (Ecdysoneless homolog) are the candidate to exhibit functional relationships with NFIC. Zhang et al. reported that CDC5L is involved in cell cycle progression, especially G2/M entry, and siRNA‐mediated silencing of CDC5L induced G2/M arrest and apoptosis of bladder cancer cells.^22^ Similarly, it has been reported that siRNA‐mediated silencing of ECD also induced G2/M arrest and apoptosis, and overexpression of ECD promoted ubiquitination and proteasome degradation of p53, a tumor suppressor protein, in gastric tumor cells.^23^ Although it remains unclear if STC‐1 is involved in the progression of lung cancer through NFIC, it is apparently worthy of further investigation in future studies.

We next investigated whether a sufficient growth‐arresting effect can be obtained by expressing suicide gene in STC‐1‐expressing lung cancer cells. Suicide genes encode enzymes that transform low‐cytotoxicity prodrugs into high‐cytotoxicity drugs. Commonly used suicide gene/prodrug combinations HSV‐tk/GCV and UPRT/5‐FU have already been applied for clinical studies of diseases such as brain tumor, ovarian cancer, and colon cancer.^24^, ^25^, ^26^, ^27^, ^28^, ^29^, ^30^, ^31^, ^32^, ^33^, ^34^ In earlier studies, some viral vectors such as adenovirus and adeno‐associated virus were used for transducing suicide genes into tumor cells. Although these virus vectors are easily manipulated, they exhibit high titer, the expression of transduced genes in target cells is transient, local, and non‐specific.^35^, ^36^ To circumvent these difficulties, the vector and strategy designs have been improved. Maemondo et al. reported that an adenovirus vector in which the E1A gene was expressed downstream of the Secretory leukoprotease inhibitor (SLPI) promoter can selectively exert lung‐cancer‐specific cytotoxicity.^37^ This study confirmed that suicide gene therapy by expressing UPRT gene under the control of STC‐1 promoter was effective to target STC‐1‐expressing lung cancer cells. The development of new strategies that deliver the STC‐1 promoter and UPRT gene construct, such as establishment of viral vectors with low cytotoxicity and immunogenicity, or injection of expression vector via bronchoscope, persist as challenges for future study.

We further evaluated whether targeting of STC‐1‐expressing lung cancer cells would influence adjacent STC‐1‐negative cells, or not. By targeting STC‐1‐positive cells, we induced a significant growth‐arresting effect toward STC‐1‐negative tumor cells and confirmed that the UPRT/5‐FU system exhibits a significant bystander cytotoxic effect both in vitro and in vivo. Although the mechanism remains unclear, Kawamura et al. and other researchers reported that it might depend on a gap‐junction‐mediated intercellular communication, in part accompanied by connexin 43.^38^ In general, solid tumors consist not only of tumor cells, but also of fibroblasts, stromal cells, tumor‐associated macrophages, and the extracellular matrix. They cooperatively promote tumor progression, invasion, and metastasis. Although it was uncertain if targeted STC‐1‐positive lung cancer cells damaged adjacent non‐tumor cells from our study, our results might also represent a promising strategy to target the tumor microenvironment. It may achieve additional therapeutic benefits that STC‐1‐targeting strategy is combined with immune checkpoint therapy, radiotherapy, or chemotherapy.

Some issues that have been raised to date must be overcome before application of STC‐1 as a new molecular target of lung cancer. Particularly because STC‐1 is a soluble molecule, it seems necessary to synthesize humanized anti‐STC‐1 neutralizing antibodies and STC‐1 antagonists. In addition, because no receptor for STC‐1 has been identified, a downstream signal pathway leading from STC‐1 to cell proliferation, anti‐apoptosis, and enhanced glucose metabolism has not been fully clarified. Nevertheless, STC‐1 knockout mice grow normally, showing no abnormality. In addition, we have preliminary data showing that the expression of STC‐1 is tend to higher in cells in anaerobic conditions than those in aerobic conditions. A common feature of most solid tumors is a low level of oxygen, called hypoxia, compared to normal tissues. For that reason, the side effects by inhibition of signaling pathway via STC‐1 are expected to be negligible. In the future, uncovering molecules as well as signaling pathways that are involved in the growth of cancer cells downstream of STC‐1 will open possibilities for further clinical applications of STC‐1.

## Supporting information

Fig S1‐S2

## Data Availability

The data that support the findings of this study are available from the corresponding author, SO, upon reasonable request.
