# Supplementary figures and images for: Targeting stanniocalcin‐1‐expressing tumor cells elicits efficient antitumor effects in a mouse model of human lung cancer
Source: Cancer Med. 2021 Apr 7;10(9):3085–100. doi: 10.1002/cam4.3852 (PMC8085941; doi:10.1002/cam4.3852)

A

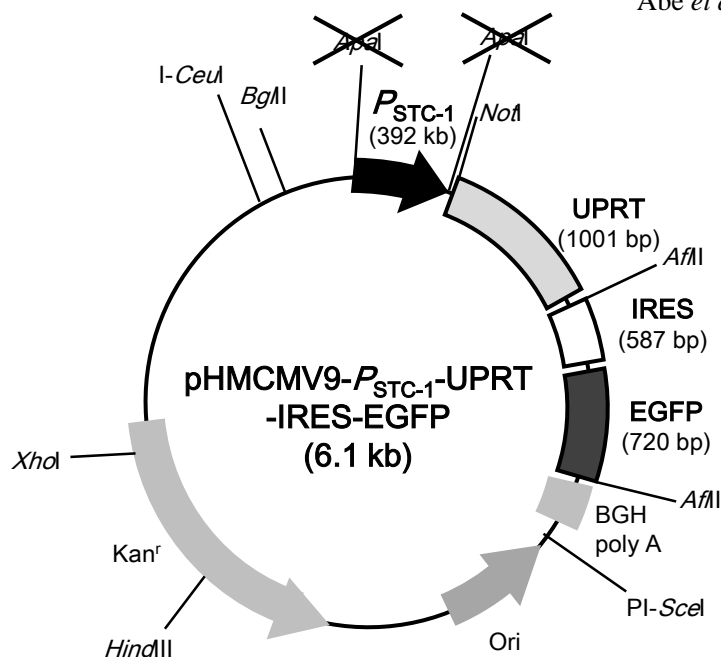

B

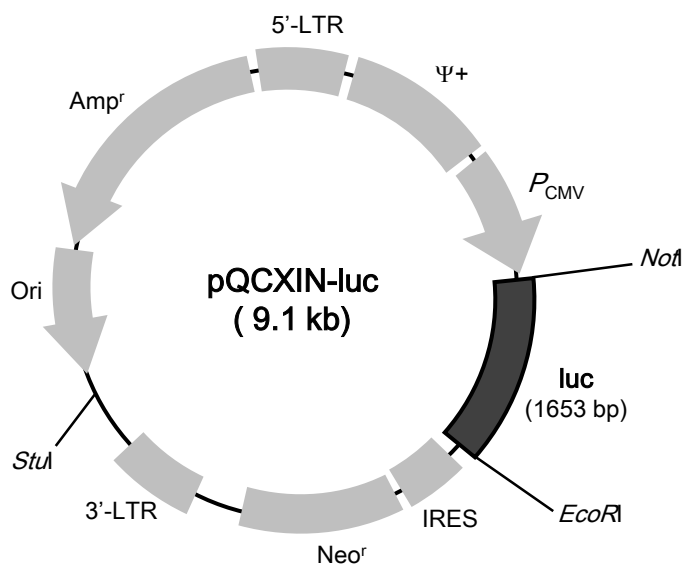

## Gating Strategy (PC-9)

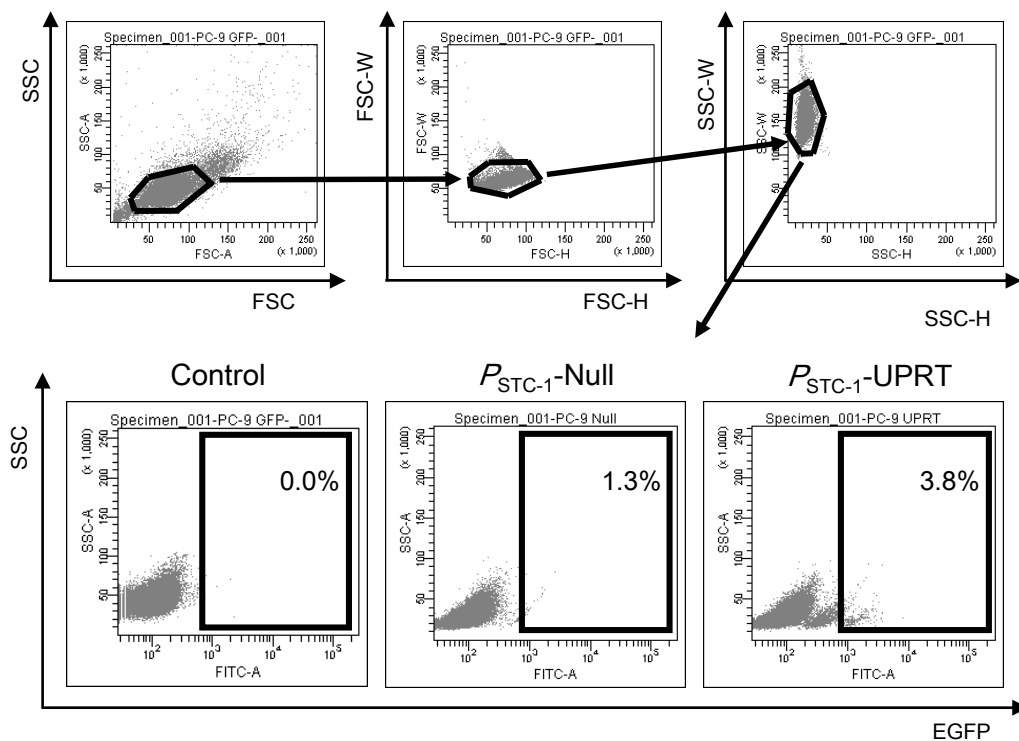

Supplement: Supplementary file 1 — Fig S1‐S2 [file CAM4-10-3085-s001.pdf]
